# Presence of Resistant *Enterobacteriaceae* in Poultry and Synanthropic Birds of an Urban Context of Social Farming in Southern Italy

**DOI:** 10.3390/vetsci12100961

**Published:** 2025-10-09

**Authors:** Antonino Pace, Mattia Longobardi, Tamara Pasqualina Russo, Luca Borrelli, Alessandro Fioretti, Ludovico Dipineto, Antonio Santaniello

**Affiliations:** Department of Veterinary Medicine and Animal Production, University of Naples Federico II, 80137 Naples, Italy; antonino.pace@unina.it (A.P.); mattlong94@libero.it (M.L.); tamarapasqualina.russo@unina.it (T.P.R.); luca.borrelli@unina.it (L.B.); fioretti@unina.it (A.F.); dipineto@unina.it (L.D.)

**Keywords:** zoonoses, avian species, antimicrobial resistance, human–animal interaction, social farming services, one health, contact risk, phenotypic resistance

## Abstract

**Simple Summary:**

Social Farming refers to a growing practice that uses agricultural and animal-related activities to support rehabilitation, social inclusion, health and education. Within these contexts, people often interact with animals; however, little is known regarding the risk of infections for users due to pathogens carried by the animals or present in contaminated environments. Therefore, this study was carried out in a Social Farming context in Naples to assess the presence of potential zoonotic enterobacteria (e.g., *Escherichia coli*, *Klebsiella* spp., *Salmonella* spp.) in both animals and on environmental surfaces. The results show that animals and surfaces in Social Farming contexts may harbor potentially pathogenic agents, most of which were resistant to a commonly used antibiotic. These findings suggest a health risk to animals and humans, especially vulnerable populations, interacting in shared environments. However, continuous monitoring, good hygiene practices, and proper animal management will help preserve the health of humans and animals involved, while supporting the valuable benefits of Social Farming.

**Abstract:**

Social Farming promotes mental and physical health, social inclusion, education and recreational services through agricultural and animal-related activities. The expansion of Social Farming draws attention to its potential health risks, although information on the role of animals and environments as reservoirs of pathogenic or resistant bacteria within Social Farming contexts is still limited. Therefore, this study aimed to assess the presence of potential zoonotic enterobacteria (e.g., *Escherichia coli*, *Klebsiella* spp., *Salmonella* spp.) and their antibiotic-resistance profiles from animals and environmental samples within a Social Farming context in Naples. Samples were collected from 76 animals belonging to 5 species and from 16 environmental surfaces. Bacteriological investigations included isolation of *Enterobacteriaceae*, identification through MALDI-TOF, and antibiotic susceptibility testing. The most frequently isolated species were *E. coli* and *Klebsiella* spp., both from animal (73.7% and 44.7%, respectively) and environmental samples (56.3% and 43.8%, respectively). Notably, 96.9% of tested strains were resistant to amoxicillin-clavulanic acid. These findings suggest that poultry, synanthropic birds and environmental surfaces within a Social Farming context might harbor potentially pathogenic and antibiotic-resistant bacteria. Thus, continuous monitoring, good hygiene, and proper management are required strategies to preserve the health of users, especially vulnerable populations such as children.

## 1. Introduction

During the World Conference on Environment and Development in Rio de Janeiro in 1992 (Earth Summit in Rio), the concept of “multifunctionality” was introduced for the first time at an international level. In Europe, this term appeared later, in the Cork Declaration, which is considered to be a real cultural revolution because it highlighted the importance of the multifunctionality of agriculture, primary sector capable of providing not only food, but also services of social inclusion, well-being and health to people.

Social Farming (SF) is a rapidly growing phenomenon in Italy and across Europe, aiming to provide the new generations and disadvantaged categories with an opportunity for training in the fields of food production, agricultural practices and rural life, as well as knowledge of animal species beyond dogs and cats. Social farming has been shown to support mental health, reduce social exclusion, and foster personal growth, especially in children and vulnerable individuals, by promoting interaction with nature and animals and by providing opportunities for education and entertainment. Furthermore, SF contributes to rural development and regional resilience, enhancing sustainability and social cohesion [1,2,3]. However, the benefits of these practices should be weighed against potential public health risks. Places where SF is practiced (Educational Farms, Social Farms, Social Promotion Associations) encourage close contact between humans and animals, which can facilitate zoonotic transmission of pathogens. Indeed, apparently healthy animals may carry pathogens transmissible to humans, including enteropathogenic bacteria (e.g., *Escherichia coli*, *Salmonella* spp., *Enterobacter* spp.) [4]. Although most strains are harmless, some strains are responsible for intestinal diseases of varying severity and extra-intestinal diseases. Outbreaks and case reports involving enteric pathogens have been documented in animal-contact venues, such as petting zoos, livestock fairs and similar public settings [5]. The most frequently implicated agents include *E. coli* O157, non-O157 Shiga toxin-producing *E. coli* (STEC), and *Salmonella* spp., with mammals being the primary animal species reported [6]. In these contexts, disease transmission can occur not only following direct animal contact but also indirectly via contaminated environments and fomites, including ingested dust or other sources (e.g., pacifiers, food, etc.) [6,7]. This is particularly concerning in SF contexts that attract many people and especially children, who commonly have less rigorous hygiene practices than adults and are more susceptible to serious disease outcomes [8].

In addition, animals can serve as important carriers of antibiotic-resistant bacteria, which represent a growing concern for both human and veterinary medicine [9]. Antibiotic resistance occurs when bacteria develop defense mechanisms to resist antibiotics, reducing the effectiveness of these drugs in treating infections [10]. The indiscriminate and inappropriate use of antibiotics in human and animal medicine is one of the main factors contributing to the rise and spread of antibiotic resistance. The use of antibiotics in livestock for therapeutic, prophylactic and growth-promoting purposes has contributed to the emergence of antibiotic-resistant bacteria in the agricultural sector [11]. Indeed, studies in petting zoos and other animal-contact venues have reported antibiotic-resistant bacteria in animals and on environmental surfaces, highlighting that resistance is an additional hazard in these contexts [6,12]. These resistant bacteria can be transmitted to humans through the consumption of contaminated food, direct contact with animals, or exposure to the surrounding environment, such as water, soil, and air [11].

The presence of wild animals carrying zoonotic pathogens or antibiotic-resistant bacteria might further exacerbate the risk within SF contexts. In particular, synanthropic birds might play a key role in SF contexts, due to their ability to thrive in peri-urban and rural areas, where they frequently forage on waste or animal feed and roost near agricultural facilities [13]. These birds may harbor both zoonotic agents and resistant bacteria, and can contaminate feed, water sources, and soil through their droppings [14,15]. This represents a significant risk in shared environments where domestic animals and humans, especially children, interact closely and might come into contact with contaminated surfaces, such as benches, enclosures, or other areas present in SF contexts.

In light of all the above, it is important to evaluate, without discouraging, the interactions that humans have with animals and the environment, considering that contact with animals can be a beneficial and pleasant activity, but not without zoonotic risks.

Furthermore, while there are numerous studies on the benefits for people and the social purposes of these types of contexts, there are no research experiences in the literature that have focused on the risks of zoonosis related to an SF context.

Therefore, the objective of the present investigation was to carry out a bacteriological survey within a Social Farming context in Naples, focusing on environmental surfaces, synanthropic and farmed avian species, to detect the presence of pathogenic enterobacteria and to characterize their phenotypic profiles of antibiotic-resistance.

## 2. Materials and Methods

### 2.1. Sampling

A total of 92 samples were collected during the bacteriological survey at a Social Farming context in the city of Naples. These included 76 cloacal samples, each obtained from a different individual bird, belonging to 5 different species present in the Social Farming context, and 16 environmental samples, each obtained from a different surface commonly in contact with animals and humans, as shown in Table 1. Each animal was sampled once using a sterile swab, subsequently inoculated into Amies transport medium (Thermo Fisher Scientific, Milano, Italy). Each environmental surface (e.g., benches, drinking troughs, feeders, fences, logs and tables) was sampled once using a sponge-bag (Thermo Fisher Scientific, Milano, Italy). Pigeons (*Columba livia*) were captured by use of specific cage traps (The Trap Man, Ormskirk, UK) baited with grains. The traps were placed, the evening before the day of sampling, in the site overlooking the area frequented by poultry. From each captured pigeon, a single cloacal sample was collected using a sterile swab, as was performed for farmed avian species. All sampled animals were apparently healthy and sampling was carried out in compliance with Ethical Animal Care and Use Committee of University of Naples Federico II.

### 2.2. Bacterial Isolation and Identification

All collected swabs were transported to the Laboratory of the Centro Sperimentale Avicunicolo (University of Naples Federico II, Italy) within three hours of collection in the field. Each swab sample was placed in Peptone Water (Oxoid, Milano, Italy), incubated aerobically at 37 °C for 24 h, and then streaked on selective and differential media. In detail, an aliquot of culture broth (10 µL) was streaked on MacConkey Agar medium (Oxoid, Milano, Italy) and incubated at 37 °C for 24 h. All colonies were subjected to morphological examination, oxidase and catalase tests and streaked on chromogenic media, specifically Tryptone Bile X-Gluc agar (Oxoid, Milano, Italy) and Brilliance Salmonella Agar (Oxoid, Milano, Italy), which were incubated at 42 °C for 24 h. The obtained colonies were cryopreserved in Brain Heart Infusion broth (Oxoid, Milano, Italy) supplemented with glycerol (20%) for subsequent identification by Matrix-Assisted Laser Desorption/Ionization Time-Of-Flight (MALDI-TOF). For isolation of *Salmonella* spp., the starting samples were enriched in selective Rappaport Vassiliadis broth (Oxoid, Milano, Italy) and incubated at 42 °C for 24 h. Subsequently, the broth cultures were streaked on Brilliant Green medium (Oxoid, Milano, Italy) and on Xylose Lysine Desoxycholate (Liofilchem Ltd., Teramo, Italy) Agar, incubated at 37 °C for 24 h. Suspect colonies, drop-shaped and not fermenting lactose, were subjected to oxidase tests and cryopreserved for identification by MALDI-TOF as previously described. As part of routine laboratory practice, reference strains (*E. coli* ATCC 25922; *K. pneumoniae* ATCC 700603, and *Salmonella typhimurium* ATCC 14028) were included as positive controls and sterile broths as negative controls during bacterial isolation. The isolates collected and cryopreserved in Brain Heart Infusion broth were streaked onto Nutrient Agar (Oxoid, Milano, Italy), incubated at 37 °C for 24 h and subjected to identification by MALDI-TOF. The instrument was routinely calibrated according to manufacturer’s instructions. The mass spectra were acquired from each isolate and were analyzed using the MALDI Biotyper 4.1.100 software (Bruker Daltonics, Bremen, Germany) according to the manufacturer’s criteria. Specifically, the software compares the spectra with the reference database and assigns a score value reflecting identification reliability: scores ≥ 2.0 are acceptable as reliable species-level identification; scores between 1.7 and 1.99 are acceptable as reliable genus-level identification; scores < 1.7 are considered not reliably identified. Differences in the prevalence of bacterial species between animal and environmental samples were assessed using Chi-square or Fisher’s Exact Test, as appropriate, and statistical significance was set at *p* < 0.05.

### 2.3. Antibiotic Susceptibility Testing

All the isolated strains were subjected to antibiotic susceptibility testing using the disk diffusion technique and in accordance with the EUCAST Disk Diffusion Method for Antimicrobial Susceptibility Testing [16]. Antibiotic disks (Liofilchem Ltd., Teramo, Italy) were selected among highly important and critically important antibiotics, according to the WHO’s List of Medically Important Antimicrobials [17], and included the following antibiotics: amoxicillin-clavulanic acid (AUG, 20/10 μg); ampicillin-sulbactam (AMS, 20 μg); aztreonam (ATM, 30 µg); cefepime (FEP, 30 μg); ceftazidime (CAZ, 30 µg); ceftriaxone (CRO, 30 μg); chloramphenicol (C, 30 µg); gentamicin (CN, 10 µg); marbofloxacin (MAR, 5 μg); norfloxacin (NOR, 10 μg); tigecycline (TGC, 15 μg); tobramycin (TOB, 10 μg). Isolates were classified as susceptible or resistant according to the EUCAST breakpoints tables for interpretation of MICs and zone diameters [18]. In addition, all strains were screened for the detection of extended-spectrum β-lactamases (ESBLs) using the combined ESBL + AmpC screen disk kit (Liofilchem Ltd., Teramo, Italy). Antibiotic resistance rates were compared between isolates from animal and environmental samples, using Chi-square or Fisher’s Exact Test, as appropriate, and statistical significance was set at *p* < 0.05.

## 3. Results

Out of a total of 92 analyzed samples (76 animal and 16 environmental samples), 116 bacterial strains were isolated (97 from animal and 19 from environmental samples), with the highest prevalence detected for the species *Escherichia coli* in both animal and environmental samples (73.7% and 56.3%, respectively), followed by *Klebsiella* spp. (44.7% and 43.8%, respectively), *Enterobacter* spp. (6.6% and 7.6%, respectively) and *Citrobacter* spp. (2.6% and 3.3%, respectively). In contrast, *Salmonella* spp. was not detected. Details of the results from bacterial isolation (i.e., number/percentage of bacterial species/genus isolated from each animal species and type of environmental surface sampled) are shown in Table 2. No significant differences were detected in the prevalence of *Enterobacteriaceae* species between animal and environmental samples.

**Figure 1:**
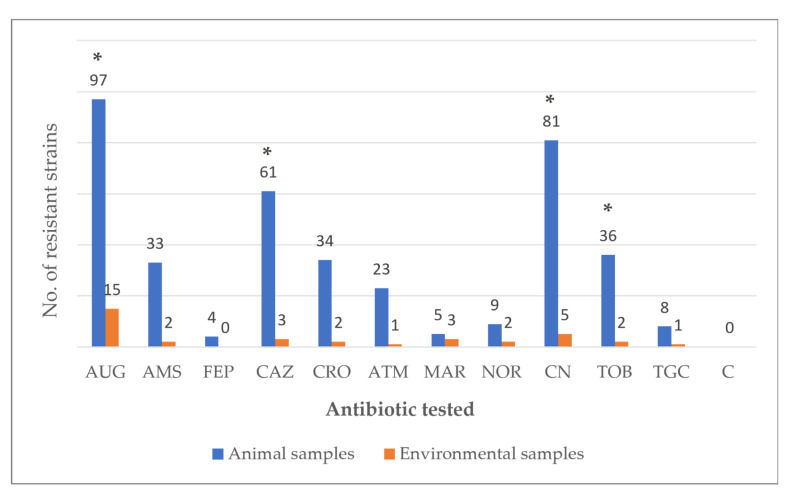
Antibiotic resistance of 116 strains of *Enterobacteriaceae*, isolated from 76 avian cloacal samples and 16 environmental samples, within a social farming context. Legend: AUG: Amoxicillin-clavulanic acid (20/10 μg); AMS: Ampicillin–sulbactam (20 μg); FEP: Cefepime (30 μg); CAZ: Ceftazidime (30 µg); CRO: Ceftriaxone (30 μg); ATM: Aztreonam (30 µg); MAR: Marbofloxacin (5 μg); NOR: Norfloxacin (10 μg); CN: Gentamicin (10 µg); TOB: Tobramycin (10 μg); TGC: Tigecycline (15 μg); C: Chloramphenicol (30 µg). *: Statistically significant difference between resistant strains of animal samples compared to environmental samples (*p* < 0.05).

Antibiotic-resistance rates were below 40% for all twelve tested antibiotics (Figure 1), except for amoxicillin–clavulanic acid with 96.6% (112/116, 97 from animal and 15 from environmental samples), gentamicin 74.1% (86/116, 81 from animal and 5 from environmental samples) and ceftazidime 55.2% (64/116, 61 from animal and 3 from environmental samples). In contrast, all strains were susceptible to chloramphenicol. No isolates positive on the ESBL + AmpC screening test were detected. When comparing resistance rates between sources (animal vs. environmental samples), a significantly higher number of resistant strains was observed in animal samples compared to environmental samples for the following antibiotics (*p* < 0.05): amoxicillin–clavulanic acid (100% vs. 78.9%); ceftazidime (62.9% vs. 15.8%); gentamicin (83.5% vs. 26.3%); and tobramycin (37.1% vs. 10.5%).

## 4. Discussion

The results of our investigation highlighted the presence of numerous enterobacteria, which are part of the common intestinal flora of numerous animal species, although some strains have been reported as relevant nosocomial pathogens, causing various infections, sometimes with serious clinical outcomes [19]. The highest prevalence, both in animal and environmental samples, was detected for *E. coli*, which is an integral part of the normal intestinal microbiota of humans and other warm-blooded animals, including avian species. Although most strains are harmless, some strains, described also in wild and domestic birds, are responsible for intestinal diseases of varying severity (which can manifest with abdominal pain, vomiting, bloody diarrhea) and extra-intestinal diseases, such as urinary tract infections, peritonitis, septicaemia, pneumonia and meningitis [14,20]. The second most frequent bacterial species belonged to the genus *Klebsiella,* detected in approximately 44% of animal and environmental samples. Whilst *K. oxytoca* is considered an emerging opportunistic pathogen [21], *K. pneumoniae* is widely recognized as the cause of serious infections (urinary tract infections, pneumonia, septicaemia, necrotizing infections, and pyogenic liver abscesses), especially among hospitalized or immunocompromised individuals [22]. The detection of *Enterobacter* spp. is also relevant because, alongside *K. pneumoniae,* this genus is part of the ESKAPE pathogens (*Enterococcus faecium*, *Staphylococcus aureus*, *Klebsiella pneumoniae*, *Acinetobacter baumannii*, *Pseudomonas aeruginosa* and *Enterobacter* spp.), which are major contributors to nosocomial infections and antibiotic resistance worldwide [23]. These bacteria are not confined to clinical settings: *K. pneumoniae* and *Enterobacter* spp. have also been isolated from wild and synanthropic birds, confirming their presence in natural environments outside hospitals [24]. This highlights the potential for environmental and wildlife reservoirs to contribute to the broader circulation of clinically relevant pathogens. More importantly, the acquisition of antibiotic resistance genes by ESKAPE pathogens has reduced therapeutic options for severe infections, increased disease burden and mortality rates due to treatment failure, and requires a coordinated global response for antibiotic resistance surveillance [25].

Enteric pathogens have been reported in both animals and their surrounding environments within animal-contact settings. For example, Conrad et al. [6] detected non-O157 STEC in 29% of avian samples and 4% of environmental samples. This prevalence is lower compared to our results, although their analysis was specific to STEC, while our study assessed *E. coli* in general, which may represent a limitation of direct comparison. However, consistent with our results, *Salmonella* was absent from all isolates [6]. Similarly, low prevalence of *Salmonella* and STEC was reported in other animal-contact venues [4,8], underlining how pathogen prevalence may vary substantially across species, contexts and geographical regions.

Our findings highlight the relevance of *Enterobacteriaceae* as major reservoirs and vectors of resistance genes in both human and animal populations. Indeed, commensal bacteria are exposed to pressures exerted on the host gut microbiota throughout their life [26]. As a result, these strains may acquire some resistant genes and/or undergo mutations that may alter the microbial homeostasis in the gut environment [27]. These changes in commensal microorganisms could represent a risk for the transfer of resistance genes to other species, so it is important to monitor the effects of antibiotic use on the development of resistance in commensal microorganisms of livestock and birds [28]. Indeed, in the present study, the percentage of resistant strains was significantly higher in animal isolates compared to environmental isolates for amoxicillin–clavulanic acid, ceftazidime, gentamicin and tobramycin. This pattern supports the hypothesis of selective pressure associated with antimicrobial use in veterinary contexts and is consistent with previous studies, reporting higher resistance rates in animal isolates compared to environmental strains [29,30].

In our study, resistance to amoxicillin-clavulanic acid was extremely high (97%), whereas studies in petting zoo animals reported lower resistance rates, with common resistance patterns involving tetracycline, streptomycin and kanamycin [6]. Only ESBL-producing *Enterobacteriaceae* recovered from 12% of animals in a petting zoo survey exhibited similarly high rates of resistance to amoxicillin–clavulanic [12]. However, their isolates were susceptible to gentamicin, in contrast with our results (showing 74% of resistance). This divergence may reflect differences in local antibiotic use and animal populations, such as the predominance of avian hosts in our study, compared with the mammalian species commonly examined in other animal-contact venues. Indeed, amoxicillin-clavulanic acid is one of the most widely used agents in many countries, mainly prescribed for respiratory and urinary tract infections [31]. The resistance to amoxicillin-clavulanic acid recorded during our study is in line with the results of a survey conducted in China [32]. These authors reported that 90% of the *E. coli* strains isolated from chickens were resistant to the combination of amoxicillin-clavulanic acid. In contrast, the results of a study conducted in Ghana [28] showed that all the strains isolated from broilers were susceptible to amoxicillin-clavulanic acid. Our result is particularly relevant because, according to the World Health Organization (WHO), the combination of amoxicillin and clavulanic acid is considered a “Highly Important Antimicrobial”. This is due to its wide use in human medicine and its importance in the treatment of various bacterial infections [17]. Another area of concern within SF contexts is represented by the presence of synanthropic birds, such as pigeons (*C. livia*), which have been identified as carriers of pathogenic *Escherichia coli* (e.g., APEC, EPEC, STEC) and other zoonotic agents, including *Salmonella* and *Campylobacter* [13,14,15,20,33]. Additionally, synanthropic birds can acquire antibiotic-resistant bacteria from environmental exposure to human and veterinary waste or contaminated feed, as evidenced by the presence of antibiotic-resistant bacteria in these species [34,35]. Therefore, their mobility and adaptability allow them to connect urban and rural areas, facilitating environmental contamination and posing a potential health risk to domestic animals and humans interacting in shared outdoor spaces. These findings underline the importance of continuous environmental monitoring and the integration of wildlife surveillance into public and veterinary health programs, in alignment with the One Health approach.

## 5. Conclusions

In conclusion, the results of this research represent the first experience in the field of Social Farming that, through monitoring the presence of potential pathogens, highlights the need to investigate the zoonotic risks of zoonosis associated with this type of context, where the reared animals can act as potential vectors and contribute to environmental spread. *Enterobacteriaceae* are a large group of microorganisms, some of which are potentially pathogenic for humans and animals (e.g., enteropathogenic *E. coli*, *Klebsiella* spp., *Enterobacter* spp.); therefore, their monitoring allows early detection of pathogenic or antibiotic-resistant strains, contributing to safeguarding the health of social farming users. Additionally, the spread of resistance towards medically important antibiotics reinforces the need for antimicrobial stewardship and prudent use in both veterinary and human medicine.

Our findings could contribute to protecting people frequenting farms from potential infections by supporting the design and application of corrective measures adapted to each Social Farming context. Since such contexts are often frequented by vulnerable groups, such as children and immunocompromised individuals, the health risk should not be underestimated. In the city of Naples, children under 14 years and individuals over 65 years represent about 15.7% and 18% of the population, respectively, confirming that vulnerable groups make up an important proportion of the community and reinforcing the need for preventive measures in Social Farming contexts. After a correct and thorough assessment and identification of zoonosis risks, corrective measures could be implemented, such as good hygiene practices, including the regular disinfection of animal and food areas, physical separation of eating areas and animal interaction zones, and careful animal management. Additionally, it may be helpful to (i) provide protective clothing; (ii) monitor contact with or handling of animals; (iii) check for the presence of vector animals; (iv) train employees about risks and preventive measures (including periodic refresher courses); (v) inform visitors about potential risks and precautions using appropriate printed materials and signage; (vi) conduct periodic (e.g., monthly) microbiological monitoring of animals, as well as environmental health and hygiene checks.

## Figures and Tables

**Table 1:** Avian species and type of environmental surfaces examined in a social farming context.

Animal Species	No. of Sampled Animals
*Anser anser*	8
*Anser cygnoides*	4
*Cairina moschata*	16
*Columba livia*	20
*Gallus gallus domesticus*	28
Subtotal	76
Environmental Surfaces	No. of sampled surfaces
Benches	4
Drinking trough	2
Feeders	2
Fences	2
Logs	4
Tables	2
Subtotal	16
Grand Total	92

**Table 2:** Prevalence of *Enterobacteriaceae* in cloacal samples of avian species and environmental samples collected in a social farming context.

Animal Species	*Citrobacter* spp.	*Enterobacter* spp.	*E. coli*	*K. oxytoca*	*K. pneumoniae*
*A. anser*	-	-	25% (2/8)	-	-
*A. cygnoides*	-	-	50% (2/4)	-	25% (1/4)
*C. livia*	10% (2/20)	25% (5/20)	85% (17/20)	-	45% (9/20)
*C. moschata*	-	-	68.8% (11/16)	37.5% (6/16)	43.8% (7/16)
*G. gallus domesticus*	-	-	85.7% (24/28)	-	39.3% (11/28)
Subtotal	2.6% (2/76)	6.6% (5/76)	73.7% (56/76)	7.9% (6/76)	36.8% (28/76)
Environmental surfaces					
Benches	-	-	50% (2/4)	-	25% (1/4)
Drinking trough	50% (1/2)	-	-	-	-
Feeders	-	100% (2/2)	50% (1/2)	-	-
Fences	-	-	100% (2/2)	50% (1/2)	-
Logs	-	-	75% (3/4)	50% (2/4)	50% (2/4)
Tables	-	-	50% (1/2)	-	50% (1/2)
Subtotal	6.3% (1/16)	12.5% (2/16)	56.3% (9/16)	18.8% (3/16)	25% (4/16)
Grand Total	3.3% (3/92)	7.6% (7/92)	70.7% (65/92)	9.8% (9/92)	34.8% (32/92)

## Data Availability

The original contributions presented in this study are included in the article. Further inquiries can be directed to the corresponding author.
